# Expression Profile and Function Analysis of LncRNAs during Priming Phase of Rat Liver Regeneration

**DOI:** 10.1371/journal.pone.0156128

**Published:** 2016-06-21

**Authors:** Jun Li, Wei Jin, Yanli Qin, Weiming Zhao, Cuifang Chang, Cunshuan Xu

**Affiliations:** 1 College of Life Science, Henan Normal University, Xinxiang 453007, Henan Province, China; 2 State Key Laboratory Cultivation Base for Cell Differentiation Regulation and Henan Bioengineering Key Laboratory, Henan. Normal University, Xinxiang 453007, Henan Province, China; 3 Henan engineering laboratory for bioengineering and drug development, Henan Normal University, Xinxiang 453007, Henan Province, China; University of Saarland Medical School, GERMANY

## Abstract

Emerging evidences have revealed that long non-coding RNAs (lncRNAs) functioned in a wide range of physiological and pathophysiological processes including rat liver regeneration, and could regulate gene expression in the transcriptional and post-transcriptional levels. However, the underlying mechanism for lncRNAs participation in liver regeneration is largely unknown. To define the mechanisms how the lncRNAs regulate LR, we performed bio-chip technology, high-throughput sequencing and RT-PCR to detect the expression of lncRNAs at 0, 2 and 6 h during LR after 2/3 hepatectomy (PH). The results indicated that 28 lncRNAs were involved in LR. Bioinformatics analysis predicated 465 co-expression target genes including 10 regulatory genes were related to these 28 lncRNAs. Ingenuity Pathway Analysis (IPA) was employed to analyze the signaling pathways and physiological activities that regulated by these genes, and the results suggested that these genes were potentially related to ILK, SAPK/JNK and ERK/MAPK signaling pathways, and possibly regulate many important physiological activities in LR in terms of cell proliferation, cell differentiation, cell survival, apoptosis and necrosis.

## Introduction

Liver is an important organ, and plays an irreplaceable role in regulating metabolism and has various functions including compound detoxification, biliary secretion, absorption, transportation, storage, hematopoiesis, regeneration [1,2]. The remnant liver will recover its original mass and volume through liver regeneration (LR) by compensatory hyperplasia, when injured after partial hepatectomy (PH)[3,4]. Prior studies showed LR after the 2/3 PH could be divided into 3 phases: priming phase (2–6 h), proliferative phase (12–72 h) and terminal phase (120–168 h) according to the gene expression pattern [5,6]. However, the clinical strategies for treating liver diseases such as liver tumor removal and liver transplantation always rely on regenerative ability of the liver. Therefore, it’s crucially significant to understand the underlying mechanism of LR after PH [7].

About 90% of the eukaryotic genomes were transcribed as non-coding RNA (ncRNAs)[8], and generating an extraordinary range of RNA with little or no coding capacity [9]. Based on transcript size, these ncRNAs could be grouped into two major classes: small non-coding RNA (sncRNA) with shorter than 200bp and lncRNA with length range from 200bp to over 100kb, which are originated from multiple loci throughout the genome and could be classified as intergenic lncRNA and intragenic lncRNAs [10,11]. LncRNA could be positioned in nucleus, cytoplasm or both. Mechanistically, LncRNA can bind DNA, RNA or proteins, positively/negatively regulate gene expression at multiple levels including transcriptional and post-transcriptional [12]. Previous studies have discovered that aberrant expression of many lncRNAs was related to different kinds of human diseases, especially cancers. Pasmant et al discovered that lncRNA ANRIL(CDKN2B antisense RNA1)is transcribed form the INK4b-ARF-INK4a gene cluster in the opposite direction [13]; Zhang *et al*. concluded that the over-expression of ANRIL had important function for the development of gastric carcinoma [14]. Huang et al. demonstrated that the up-regulation of ANRIL was related to the tumor volume in hepatocellular carcinoma [15].Yamaguchi *et al*. reported that lncRNA-HEIH could promote the growth of tumors [16,17]. Recent studies claimed that various lncRNAs were involved in the process of LR, while the molecular mechanism of lncRNAs in regulating the development and process of LR is still unclear.

To understand the mechanism how the lncRNAs regulate LR, which lncRNAs expression significantly altered in LR and their potential target genes. We performed biochip technology, high-throughput sequencing and RT-PCR approaches with the liver tissues collected at different time point of 2, 6 h after PH. The results demonstrated that lncRNA may regulate LR via interacting with their target genes in different signaling pathway.

## Materials and Methods

### Ethics statement

All rats were obtained from the Experimental Animal Center of Henan Normal University, and the animal experiments were carried out in strict accordance with the recommendations in the Guide for the Care and Use of Laboratory Animals of the National Institutes of Health. And protocols were approved by Institutional Animal Care and Use Committee of Henan Normal University in China (Permit Number: SYXK2008-0105). All animal procedures were conducted in strict compliance with the current Animal Protection Law of China and all experiments were performed in strict accordance with institutional guidelines for the care and use of laboratory animals. All surgery was performed under sodium pentobarbital anesthesia, and all efforts were made to minimize suffering.

### Preparation of rat LR model

In the study, the adult male Sprague-Dawley (SD) rats, weighing 230±20g, were obtained from animal center of Henan Normal University. The rats were housed in the room of 21±2°C, relative humidity 60±10%, illumination 12 h/d (8:00–20:00) with free access to water and food. A total of 30 rats were randomly divided into 5 groups with six rats in each, including 2 partial hepatectomy (PH) groups, 2 sham-operated (SO) groups and one normal control (NC) group. The rats in PH groups were subjected to two-thirds hepatectomy following the method of Higgins and Anderson (1931). SO group received the same procedure as for the PH group without liver removal. After operation, the abdominal cavities of the rats were opened at 0, 2, 6 h to collect the liver tissues.

### High-throughput sequencing of RNA

Total RNA was extracted from the frozen mixed middle parts of the right liver lobes from all rats of each time point using a Trizol mini kit (Invitrogen Corporation, Carlsbad, California, USA) and purified following the RNeasy mini protocol (Qiagen, Inc, Valencia, CA, USA). The quality of total RNA was assessed by optical density measurement at 260/280 nm and agarose electrophoresis (180 V, 0.5 h). The sample was regarded as qualified sample, when 28S RNA to 18S RNA in it is equal to 2:1. Random hexa was used to synthesize the first strand of cDNA. The second strand was synthesized by using the first strand of cDNA, buffer, dNTPs, RNase, DNA polymerase. The cDNA product was purified following the protocol of QiaQuick PCR kit. The second strands were degraded by UNG (Uracil-N-Glycosylase) after repairing the ends, plusing A bases and sequencing connectors. And then the fragment size was selected by the agarose gel electrophoresis, and built the cDNA sequencing library by PCR. Then the cDNA sequencing library was sequenced by Illumina HiseqTM 2000 (Ou Yi Biomedical Technology Co. Ltd in Shanghai). The quality of the original data was evaluated with FASTQC software, and fragment with low quality was removed by NGS QC TOOLKITv2.3.3 software.

### Estimating the sequencing data and preprocessing

Ways of preprocessing the sequencing data quality. Before analysis, the original data was processed using the software, FastQC to estimate the quality. Then through data preprocess, sequences with low quality were wiped out using the software, NGS QC Toolkit v2.3.3. The procedure includes: wipe out reads in low quality, and threshold value of quality and length was 20, 35bp respectively; wipe out base in low quality and the threshold value was 20; excise the reads with N part in the sequence and the threshold value was 35bp.

### Reference transcriptome and genome alignment

Reference transcriptome and genome alignment to local reads. Using Tohat2 to align clean reads with rat reference transcriptome, which was downloaded into the Ensembl database, the edition of the genome sequence as Rn5.0), and the procedure was following. Firstly, align reads with the known transcriptome, and mapped reads were projected to the reference transcriptome according to the known transcriptome; then align the remnant reads with the reference transcriptome in the whole segment; last align reads segment back to the reference transcriptome. And the align method of Tohat2 included transcriptome mapping (optional), genome mapping, discovery of novel transcripts including indels and “optional” fusion points, novel transcriptome mapping and re-alignments of reads that overlap with introns. Because the annotation rat genome was comparatively complete, In this procedure, alignment of the known transcriptome first, then reference transcriptome aims to improve the precision.

### The transcriptome re-establishment and assembly analysis

In this part, based on probability model, the samples’ transcriptome was re-established under the result of each sample mapping through the cufflinks software. For the data of the special strand library, it will be accurate to analyze the strand direction where the transcriptome lies in. For the program of multiple samples, the re-established transcriptome of every sample can be merged and summarized to be a transcriptome congregation.

### Finding out the underlying long non-coding RNA

Long non-coding RNAs lack of the ability to code protein, the length of which is over 200bp. According to their local relationship with the coding strand, they are divided into intergenic lncRNA(lincRNA), intronic lncRNA, anti-sense lncRNA, sense-lncRNA, bidirectional-LncRNA and etc. Especially, the lincRNA is the bid part of total LncRNAs. While in this program, the first three kinds were screened. Based on the feature of lncRNA, three rigorous steps were used to pick out the candidates. Before screening the wanted lncRNA, it needed to figure out type and number of rat genome annotation transcript, and in the genome, there were annotation of part lncRNA or underlying lncRNA(lincRNA, sense-intronic, antisense). So with the methods as follows, the underlyinglncRNA acquired can be as novel lncRNA. 1. Alignment of the sample transcripts with reference-transcripts, then wipe out the known coding transcripts and new transcripts of the known gene locus. This step compared merged-transcripts with reference-transcripts one by one and eliminate one that totally match with or similar with already known non-lncRNAs, other ncRNAs, mRNAs using cuffcompare software. Meanwhile make sure the local types of the remnant transcripts. 2. The transcripts screened in the first step were screened again under the standard that the length is over 200bp, and the number of exons was over or equal. 3. Analyze transcripts screened in the second step, and predict the CPC coding ability of them, then eliminate transcripts with coding potential.

### Analysis of characteristic of LncRNA

The statistic of predicted lncRNAs types, intergenic lncRNA (u), intronic LncRNA(i), anti-sense LncRNA(x). We used the character u, i, x as intergenic lncRNA, intronic LncRNA, anti-sense LncRNA respectively.

### Analysis of lncRNA and mRNA expression abundance

The expression abundance of lncRNA and mRNA was calculated by RPKM (Reads per Kb per Million reads) algorithm.

Calculation formula was $RPKM=\frac{10^{6}C}{NL/10^{3}}$. Wherein, RPKM represents the expression quantity of transcripts, C represents the reads number of the only compared to the related transcript, N represents the total reads number only compared to the whole transcripts. L represents the number of the bases of the transcript. RPKM algorithm can eliminate the effect of transcripts lengths and sequencing differences on the transcript expression levels.

### Differential expression and function enrichment analysis of mRNA

In this study, using the DESeq, mRNA differential expression was detected. The genes or transcripts were determined as differential genes or transcripts under the condition that the absolute value, log2 (FC) of differential expression fold change was over 1, meanwhile pvalue ≤0.05. Then combined with GO and KEGG annotation information, the function enrichment of differential genes or transcripts having coding-protein ability was made to find out related function or signal pathways with DESeq. And they had nonrandomized relationship with differential genes or transcripts (p-value ≤0.05).

### Identification of LncRNA related to rat LR

The lncRNAs whose expression abundances were ≥20 were picked out, and the normalized signal values of in experimental groups (including PH groups and OC groups) to that in the NC group were used to calculate the ratio value or fold change. When the ratio value ≥2 or ≤0.5, it meant that lncRNAs had significant changes and when 0.5–2, it meant biologically insignificant. In order to reduce the analytical error of microarray, this study detected each sample at least three times and regarded the average value of three relative values as a credible value. In addition, only the genes, which significantly changed at least at one time point during LR with significant difference (P≤0.05) or extremely significant difference (P≤0.01) between PH and NC groups were referred to as LR-associated genes. And the candidate lncRNAs were shown in the S1 Table.

### Real-time polymerase chain reaction (qRT-PCR)

To validate the reliability of High-throughput sequencing data, the expression level of 2 lncRNAs and target genes of hepatocytes was examined by qRT-PCR. Their primers were designed using Primer Express 5.0 software, and the lncRNAs and primers used in this study were shown in S2 Table. Their first chain of cDNA was synthesized by SuperScript II RT reverse transcription system (Promega, USA). The PCR were performed by the conditions with Sybr Green I: 2min at 95°C, followed with 40 cycles for 15s at 95°C, 15s at 60°C, and 30s at 72°C. Each sample was performed in triplicates. All samples were normalized to GAPDH to calculate relative lncRNA and mRNA concentrations.

### Analysis of the targeted genes of lncRNAs and mechanism of lncRNAs interacting with target genes

Previous Studies have shown that the vast majority of functional lncRNAs are trans-regulators. LncRNAs could positively/negatively regulate gene expression in multiple layers including the epigenetic, transcriptional and post-transcriptional mechanism via a variety of ways. In detail, they could regulate gene expression both in cis (neighboring genes on the same chromosome) and in trans (distantly located genes). (1) The selection of the co-expression target genes (trans-acting). The lncRNAs and genes expression differential in the different three time points (0 h, 2 h, 6 h) were analyzed, and the gene with the absolute value of correlation (the relationship of the specific lncRNA and the gene expressed differentially) over 0.8 and p-value (the correlation verifying) less than 0.05 were considered as the co-expression target gene of the lncRNA. Then functional enrichment analysis for the target genes of the each lncRNA were analyzed by using Gene Ontology (GO) (www.geneontology.org) and KEGG (http://www.genome.jp/kegg/), and the function of target genes were used to predict the roles of the specific lncRNA. (2) Analysis of the cis-acting mechanism of the target genes and lncRNA. LncRNA may regulate its vicinal genes, therefore the encoding genes on chromosome coordinates within the range of the upstream-and-downstream 300kb were pick out and the lncRNA targeted genes were confirmed as above methods.

### Analysis of the pathways and the physiological activities in which the lncRNAs-target genes involved

Associated biological functions and predominant canonical pathways in which the LncRNAs target genes were analyzed by Ingenuity Pathway Analysis (IPA) version 9.0 (Redwood City, CA, http://www.ingenuity.com) software [18]. Briefly, these genes and corresponding extremum of expression values was firstly uploaded into “Dataset Files” of IPA software. Then the genes were performed “core analysis” in IPA. Canonical pathways and biofunctions were identified from the IPA library based on their significance to the dataset (Fisher’s Exact Test *P*-value), and in which these genes involved were looked through the “Overlay →Canonical Pathway” frame. The pathways that exceed a certain Z-score cutoff were considered pathway activation or inhibition.

## Results

### Analysis of LncRNA and mRNA expression of samples in different times

**Fig 1:**
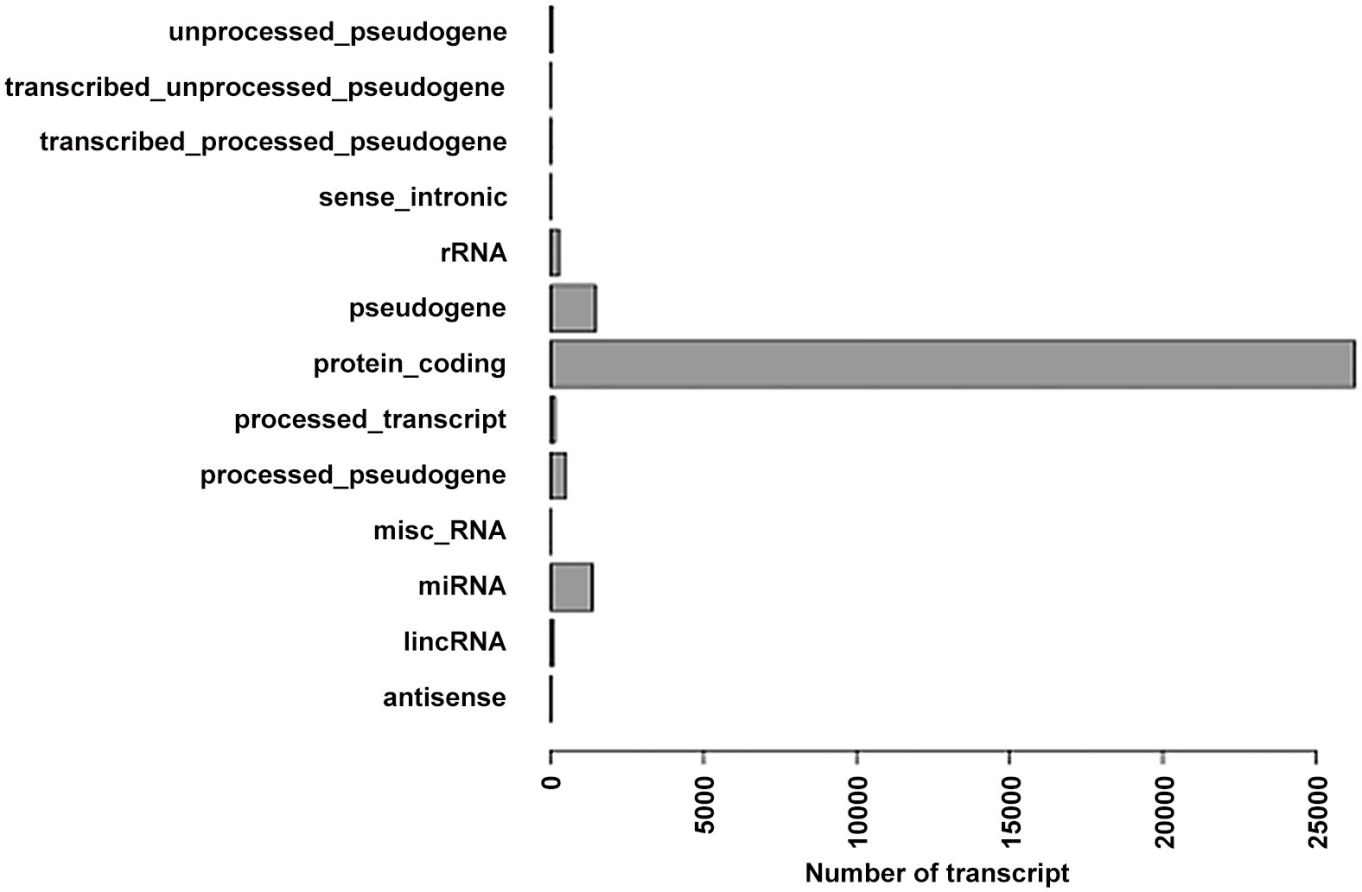
The already known transcripts of rat genome.

Because it was needed to compare the re-established transcripts with the annotation transcripts of the reference genome for screening candidate lncRNA, the type and number of annotation transcripts of the reference genome should be clear(Fig 1).

**Fig 2:**
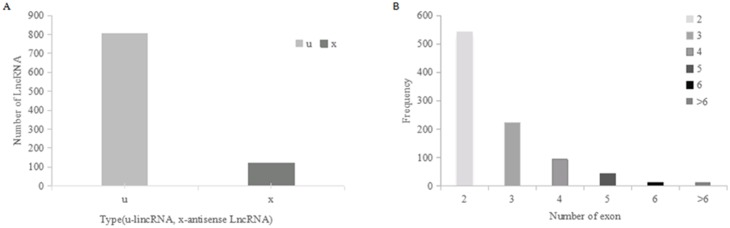
Analysis of LncRNA feature. (A) Summary of LncRNA type. (B)The exon number of LncRNA.

In Fig 1, it showed that there existed annotation of some lncRNAs or underlying lncRNAs (lincRNA, sense-intronic, antisense). So in the procedure of finding the underlying lncRNAs, the candidate lncRNAs acquired can be novel lncRNAs. In the procedure of screening LncRNA, the candidate can be found and then the feature of lncRNA was analyzed. It showed that in the determination of new lncRNAs, there are two types including lincRNA, the number of which is 804, and anti-sense lncRNA, the number of which is 122.while new lncRNAs originated from intron is not found (Fig 2A). Furthermore the lncRNAs length distribution was detected; the study found that the minimum, maximum and the average of lncRNA length are 201, 25860 and 2058(Fig 2B). So it had a bid span of length. Then exon in lncRNAs was determined and it showed that there was various difference in the exon number of lncRNAs(Fig 2B).

### Expression profiles of LncRNAs during rat LR

High-throughput sequencing was used to detect the expression profiles of lncRNAs. In total, 920 lncRNAs were found, in which 770 lncRNAs have meaningful changes compared with control group (CG, 0 h), and of them 28 lncRNAs significantly changed, 13 lncRNAs upregulated, 12 downregulated and 3 up/down regulated. More specifically, 911 lncRNAs were detected at 2 h after PH, of which 522 have meaningful changes compared with CG, among them 14 significantly changed, and 908 at 6 h, among which 680 have significant changes compared with CG, of them 16 differential changed (Table 1, S3 Table).

**Table 1:** The statistics of LncRNAs in the liver regeneration at different times.

Number of LncRNAs	PH 2h	PH 6h	In total
Investigated	911	908	920
Expressed meaningfully	522	608	770
Expressed differentially	14	16	28

### LncRNAs target genes during rat LR

**Fig 3:**
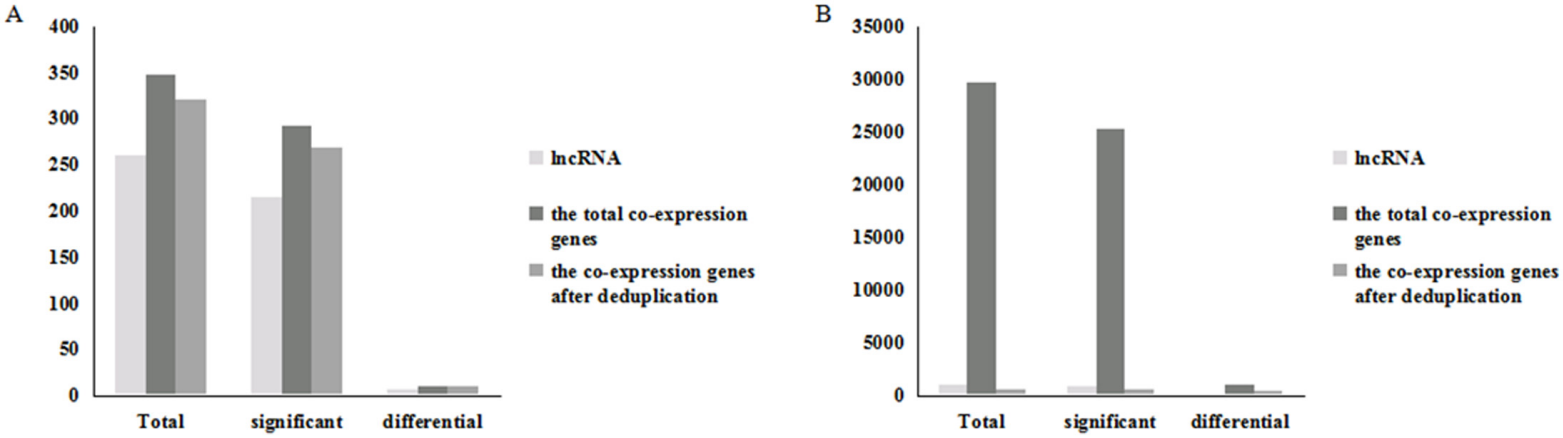
Number of the LncRNA and the co-expression or regulated genes. (A) Numbers of the lncRNAs and the co-expression genes (Trans-acting). (B) Numbers of the lncRNAs and the regulated genes (Cis-acting).

Basically, the main functions of lncRNAs were to bind specific genomic sites and ‘guide’ chromatin modifying complexes to induce epigenetic changes and regulate gene expression in the manner of cis (on neighboring genes of the same chromosome) or trans (on distantly located genes of the same or different chromosome). High-throughput sequencing was used to detect the expression changes of genes in LR. The later analysis was predicated to study the correlation between expression changes of gene and lncRNAs. Results demonstrated that 465 co-expressed (trans-acting) genes were related to the 28 differential lncRNAs (Fig 3A).

According to the cis-acting mechanism of lncRNAs, encoding genes on chromosome coordinates within the range of the up-and-downstream 300kb of lncRNAs were picked out and considered as their potential target genes. The results showed that 320 putative genes were related to 259 lncRNAs correspondingly. More specifically, 268 putative genes were related to 215 lncRNAs corresponding which have significant expression changes during LR, and 10 genes to 7 lncRNAs which have differential expression changes (Fig 3B). And the expression of these co-expression genes and regulated genes of the above lncRNAs were different from each other (Table 2).

**Table 2:** Numbers of the regulated and the co-expression genes that related to the LncRNAs.

LncRNA-ID	co-expression genes				regulated genes				
	up-regulated	down-regulated	up and down-regulated	summary	up-regulated	down-regulated	up and down-regulated		summary
TCONS_00002062	61	11	3	75	0	0		0	0
TCONS_00007963	0	0	14	14	0	0		0	0
TCONS_00008760	0	0	22	22	0	0		2	2
TCONS_00013937	7	33	0	40	0	0		0	0
TCONS_00013940	17	0	0	17	0	0		0	0
TCONS_00016913	21	1	0	22	1	0		0	1
TCONS_00022998	18	28	0	46	0	0		0	0
TCONS_00023000	16	15	0	31	0	0		0	0
TCONS_00027839	13	24	0	37	0	0		0	0
TCONS_00027980	12	19	0	31	0	1		0	1
TCONS_00032964	15	2	0	17	0	0		0	0
TCONS_00042303	23	8	0	31	2	0		0	2
TCONS_00049155	16	0	0	16	1	0		0	1
TCONS_00049316	67	11	2	80	0	0		0	0
TCONS_00053486	15	29	0	44	0	1		0	1
TCONS_00053726	9	28	0	37	0	0		0	0
TCONS_00059947	18	0	0	18	0	0		0	0
TCONS_00063525	33	16	0	49	1	1		0	2
TCONS_00063645	12	12	2	26	0	0		0	0
TCONS_00063728	67	8	2	77	0	0		0	0
TCONS_00063766	62	11	0	73	0	0		0	0
TCONS_00065281	17	31	0	48	0	0		0	0
TCONS_00066102	22	30	0	52	0	0		0	0
TCONS_00066104	0	0	13	13	0	0		0	0
TCONS_00068644	33	16	0	49	0	0		0	0
TCONS_00068955	18	0	0	18	0	0		0	0
TCONS_00068956	11	20	0	31	0	0		0	0
TCONS_00068957	11	14	0	27	0	0		0	0

### Reliability of the high-throughput sequencing detected results

Two lncRNAs (TCONS_00027980 and TCONS_00042303) and target genes which may play important roles in the LR were picked out, and their expression profiles at 0, 2 and 6 h after PH were confirmed by using Real-time PCR. Even though the abundance relative expression values of the genes detected by above two methods were not all the same, expression trends were generally consistent, suggesting that the array detection results were reliable.

### The relationship between 28 LncRNAs and target genes

**Fig 4:**
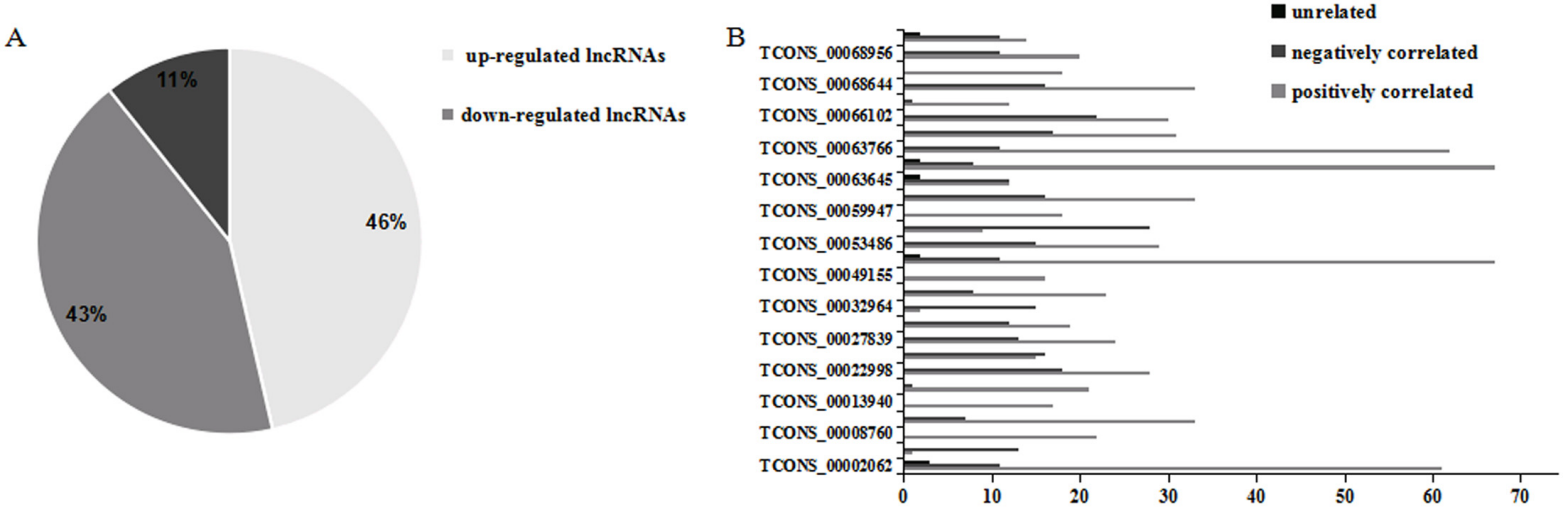
The expression of 28 lncRNAs and target genes. (A) The expression of 28 LncRNA which have significant changes. (B) The relationship between significantly expressed lncRNAs and their target genes during rat liver regeneration.

The specific expression relationship of the lncRNAs and their predicted target genes was investigated and the result showed that there were three different cases, the expression changes condition of some target genes were accordance with the lncRNAs, some contrary, while the remaining irrelevant. According to the above, the different expression of 28 lncRNAs was shown that they may regulate LR process in various ways (Fig 4A). Then the experimental results assert that the expression level of target genes could be up-regulated or down-regulated by lncRNAs (Fig 4B). The relationship between lncRNAs and target is complicated: some lncRNAs regulated one gene expression, while other could regulate multiple genes expression. Multiple lncRNAs played important roles in the LR by regulating the expression of target genes and affecting the transcription, translation, post-translation modification of proteins and other complex activities.

### The physiological activities that regulated by LncRNAs

IPA software was used to analyze the functions of 28 LncRNAs in rat LR, and the results showed that 15 LncRNAs were related to cell proliferation, 11 to cells migration, 8 to cell differentiation, and there were minorities related to cell survival, cell viability, apoptosis, necrosis, and cell death, which indicated that these LncRNAs may regulate the progression of LR via regulating cell proliferation, cell differentiation, apoptosis, etc (Table 3).

**Table 3:** The physiological activities that regulated by LncRNAs.

lncRNA	proliferation of cells	cell viability	cell survival	cell cycle progression	migration of cells	cell movement	differentiation of cells	apoptosis	nerosis	cell death
TCONS_00002062	**√**	**√**	**√**		**√**		**√**			
TCONS_00013940			**√**							
TCONS_00016913				**√**			**√**			
TCONS_00022998	**√**									
TCONS_00023000	**√**									**√**
TCONS_00027839								**√**		
TCONS_00027980	**√**				**√**	**√**	**√**			
TCONS_00042303	**√**			**√**			**√**	**√**	**√**	**√**
TCONS_00049115		**√**					**√**			
TCONS_00049316		**√**	**√**		**√**		**√**	**√**	**√**	**√**
TCONS_00053486	**√**					**√**				
TCONS_00063525	**√**				**√**	**√**		**√**	**√**	**√**
TCONS_00063645	**√**	**√**		**√**	**√**		**√**	**√**		**√**
TCONS_00063728	**√**	**√**	**√**		**√**			**√**		**√**
TCONS_00063766	**√**	**√**			**√**		**√**	**√**	**√**	**√**
TCONS_00065281	**√**				**√**	**√**				**√**
TCONS_00066102					**√**	**√**				
TCONS_00068644	**√**				**√**	**√**		**√**	**√**	**√**
TCONS_00068955	**√**									
TCONS_00068956	**√**									
TCONS_00068957	**√**			**√**	**√**	**√**	**√**	**√**		**√**

### The functions of the lncRNAs-target genes during the rat LR

IPA software was use to analyze the location of the predicted target genes, and IPA, Gene Ontology (GO) and KEGG were used to the functional annotation of the predicted target genes. The results indicated that these proteins encoded by the target genes located in the nucleus, the cytoplasm, the cell membrane, extracellular space, etc. They functioned as enzymes, proteases, kinases, transcriptional regulators, transmembrane receptors, G protein chaperone receptors, cytokines, etc via combination with other proteins, and involved in cell growth, proliferation, differentiation, apoptosis, metabolism of nucleic acids, the stress response, process development, cell communication, signal transduction, organ development, and regulating the expression of other genes controlling protein synthesis, signaling transmission in cells thereby affecting physiological activities of bodies, inflammatory responses caused by body injuries and intruders, etc. Therefore, in the LR process these genes might participate in inflammation against the organ damages, immune response, stress response, cell communication, signaling transduction, nucleic acid metabolism, organ development, molecular transport, cell proliferation and cell differentiation and other activities.

### Signaling pathways and physiological activities in which lncRNAs-target genes were involved during rat LR

**Fig 5:**
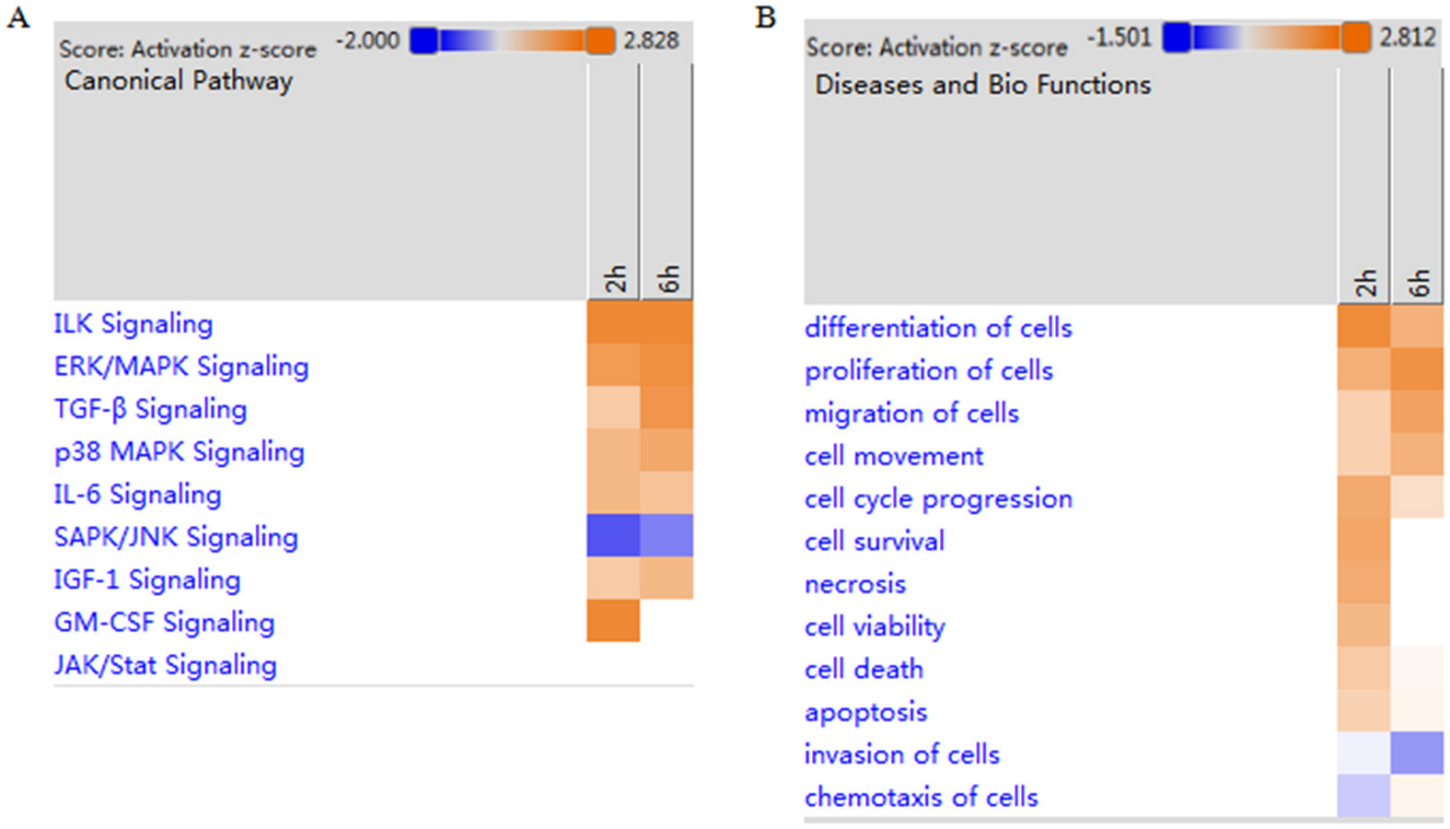
The heat maps of signaling pathways and biological functions in which significantly expressed genes were involved in rat liver regeneration. (A) The heat map of the target genes-related signaling pathways. (B) The heat maps of target genes-related biological functional.

The pathways in which lncRNAs-target genes involved were analyzed by Ingenuity Pathway Analysis (IPA) Software, and the results indicated that ILK, SAPK/JNK, TGF-β, GM-CSF, p38 MAPK, JAK/STAT, IL-6 and TGF-1 signaling pathway were predicated to be involved in lncRNA-target genes. ILK, TGF-β, GM-CSF, p38 MAPK and IL-6 signaling pathways were activated at 2 and 6 h after PH, SAPK/JNK signaling pathway was suppressed at 2 and 6 h, while JAK/STAT and TGF-1 signaling pathways were suppressed at 2 h (Fig 5). These signaling pathways were involved in many physiological activities.

## Discussion

The liver of vertebrates has a wide range of functions, as a metabolic organ, including metabolism, detoxification, glycogen storage, and protein synthesis. However, the liver is vulnerable to the internal or external injuries, such as viruses, bacteria, alcoholism, etc. and has a tremendous potential of regeneration in response to injury and stimuli [19–21]. Therefore liver regeneration plays critical roles in the clinical treatment. It is a very complex and well-controlled process, and requires participation of all mature liver cell types with hepatocytes being the main players. Immediately following surgery, growth factors and cytokines work together to induce mature hepatocytes to re-enter cell cycle, which in turn triggers cell proliferation of the other cell types in the liver. The liver mass and function is fully restored in approximately 10 days and liver mass is precisely controlled. A cascade of robust transcription regulation triggered by cytokine and growth factor signaling regulates this well orchestrated biological process [4,19,22–24]. Fortunately there has been great advance in exploring of the mechanisms of liver regeneration [21]. In recent years, the discovery of lncRNAs has dramatically altered our understanding of gene regulation and the biology of complex diseases including cancers. Recently, numerous studies have revealed that lncRNAs are dysregulated expression in various cancers [25,26]. Hu et al. discovered that as an oncogenic lncRNA, FAL1 was associated with the expression of BMI1 and repressed the expression of p21 in cancer via regulating functional genomic approach [27]. Panzitt et al. was the first to confirm that lncRNA was specifically up-regulated in hepatocellular carcinoma (HCC), HULC (highly up-regulated in liver cancer, HULC)[28]. However, research about the lncRNA regulating liver regeneration has not been fully explored. In the recent study, Lulu Huang et al. found a novel lncRNA, LncPHx2 (Long noncoding RNA induced by PHx 2), was highly upregulated during liver regeneration. Depletion of LncPHx2 during liver regeneration using antisense oligonucleotides led to a transient increase in hepatocyte proliferation and more rapid liver regeneration [29]. So to understand the relationship between lncRNAs and rat liver regeneration, the expression of lncRNAs were detected at different time points, and the result showed that the expression of 28 lncRNAs were obviously differing from the control group (0 h). For the experimental group (2 h, 6 h), the whole number of lncRNAs involved in the regulation at 2 h after PH were more than that at 6 h, while the number of the significantly and differentially expressed lncRNAs at 6 h was more than that at 2 h. The results implied that lncRNAs that involved in each stage of LR may be different, meanwhile, they positioned in different cells, different cell sites, and their functions were also different. In conclusion, different lncRNAs in different locations could regulate rat LR in various methods, the positive and negative regulation of lncRNAs leading to the highly organized of the liver regeneration process. Moreover, among the 28 lncRNAs, 13 lncRNAs were up-regulated, 12 down-regulated, 3 up /down-regulated, which may implicate that some lncRNAs could positively regulate LR directly or indirectly, while some negatively regulate.

The cis-acting, trans-acting target genes, co-expression and regulated genes of lncRNAs were predicted and picked out according to the function mechanism of lncRNAs. Previous research had demonstrated that the relationship between lncRNAs and genes were complicated and lncRNAs could regulate the expression of genes positively or negatively during the development and progression of many diseases [30–33]. And the results indicated that their expression were markedly diverse from each other and different from lncRNAs. It might suggest that lncRNAs has various methods to regulate the expression of genes during LR. The loci on chromosomes of cis-acting genes were as well aslncRNAs were searched. Previous research demonstrated that in most cases, lncRNAs exert their function by binding to various RBPs, such as WDR5 [34], GADD45A [35] and hnRNPK [36]. PRC2 was a critical regulator of histone modification, which catalyzes the trimethylation of H3K27 to mediate gene silencing. Recent findings showed that PRC2 was an important driver of tumor development and progression by suppressing various key genes, such as CDH1, DKKI and INK/ARF45. Increasing evidence has shown that many lncRNAs [37,38] were associated with PRC2 and mediate H3K27 trimethylation at distinctive target loci. Therefore, it is likely that some of 28 lncRNAs could regulate genes expression via associating with RBPs. RT-real time PCR was used to validate the expression of selected lncRNAs and their target genes. However, the result showed some lncRNAs did not change obviously, even some could not be detected, so there may play roles in different ways suggesting diverse mechanisms of lncRNAs in LR.

The signaling pathways related to target genes and lncRNAs were analyzed by using IPA software, and the results indicated that these genes have close relationship with cell proliferation, cell cycle and cell differentiation. It is likely that lncRNAs could regulate LR process through the signaling pathways that related to cell proliferation, cell differentiation, cell viability, apoptosis and other important cell activities, which hints that lncRNAs play essential roles in LR process. Recently, many studies claimed that lncRNAs could promote or inhibit the expression of target genes by elevating or descending the activities of signaling pathways [30,39–41]. Previous studied also showed that LncRNA-urothelial cancer associated 1 (UCA1) was highly expressed in bladder cancer tissues and cells, and it has been shown to play an important role in regulating aggressive phenotypes of bladder cancer cells. It enhances bladder cancer cell migration and invasion in part though hsa-miR-145/ZEB1/2/FSCN1 pathway [42]. Jiao et al. stated that in response to tissue injury, cytokines and chemokines are stimulated, which result in the activation of some downstream transcription factors (TFs) including nuclear factor-κB (NF-κB), activator protein 1 (AP-1), signal transducers and transcription activators 3 (STAT3) and CCAAT enhancer-binding protein (C/EBP), and a large number of genes including promitogenic genes and homeostatic response genes [21]. Previous research asserted that hepatocytes were activated at 2 h of LR after PH, and their transition from G0 phase to cell cycle occurred at 2–6 h [43] ILK pathway could activate the nuclear translocation of β-catenin and enhanced the transcription of LEF-1 through integrin-linked kinase pathway, and then increase the activity of Ap-1 which could promote cell proliferation [44,45], and the expression of critical cytokine IL-6 [46,47] which could affect cell proliferation via Stat3 signal pathway. It has demonstrated that transforming growth factor-β (TGF-β) signaling plays a key role in progression and metastasis of HCC [48,49], and it influences the expression of smad4, smad2/3, and β-catenin proteins and regulates cell activities [49], which indicates that it is important in liver cells. McCubrey et al. pointed out that ERK / MAPK pathway, as the most classic pathway in mitogen-activated protein kinases (MAPKs) pathways [50], had significant function in cells [51], and played a key function in tumor cell proliferation, differentiation, survival, migration, and angiogenesis [52]. It was inferred that lncRNAs above and their target genes could regulate the initiation progression of LR via regulating various signaling pathways and physical activities. In this study, some lncRNAs and their underlying target genes in the early stage of LR were selected and picked out, and the mechanism of lncRNAs and indicated potential ways to modulate genes expression were also analyzed by many methods. The rat LR was a complex progress, and it is yet to be clarified that how the specific lncRNAs could regulate rat LR. Consequently, how will the predicted target gene play role in LR and in the pathological liver disease by using tissue-specific gene overexpression and depletion approach is our next step study.

## Supporting Information

S1 Table Candidate-lncRNA. (XLSX)

S2 Table LncRNAs and target genes primers used in RT-PCR. (DOC)

S3 Table Expression profile of lncRNAs during rat LR. (XLSX)
