# Supplementary material for: Expression Profile and Function Analysis of LncRNAs during Priming Phase of Rat Liver Regeneration
Source: PLoS One. 2016 Jun 21;11(6):e0156128. doi: 10.1371/journal.pone.0156128 (PMC4915705; doi:10.1371/journal.pone.0156128)
Supplement: S2 Table — (DOC) [file pone.0156128.s002.doc]

**Supplementary Tables 1.** LncRNAs and target genes primers used in RT-PCR

| **Primers Seqname Sequences** |
| --- |
| TCONS_00027980F 5’TCCCTATACAGCCTCATAGCAATCT3’  TCONS_00027980R 5’CAAGCAATCGAACCCACTACCAC3’  TCONS_00042303F 5’TCAGGGACCTACACTCCTGTTC3’  TCONS_00042303R 5’AGGCTGATGCTTGGCTATTCT3’ |
| SIK1F 5’CAACCTGGGCGACTACAACG3’  SIK1R 5’GGAAAGGGTCACATGGGAGAAT3’  Ppp1r3bF 5’TGGCAGCGAGGAGGACAAC3’ Ppp1r3bR 5’AGCAGCGACACCGTGGAAA3’  GAPDH(RAT)F 5’CACGGCAAGTTCAACGGCACAGTCA3’  GAPDH(RAT)R 5’GTGAAGACGCCAGTAGACTCCACGAC3 |
